# The Effect of GFRP Powder on the High and Low-Temperature Properties of Asphalt Mastic

**DOI:** 10.3390/ma16072662

**Published:** 2023-03-27

**Authors:** Tao Zhen, Pinxue Zhao, Xing Zhang, Wei Si, Tianqing Ling

**Affiliations:** 1 School of Civil Engineering, Chongqing Jiaotong University, Xuefu Avenue 66, Chongqing 400074, China; 2 Sichuan Expressway Construction & Development Group Co., Ltd., Chengdu 610047, China; 3 Key Laboratory for Special Area Highway Engineering of Ministry of Education, Chang’an University, Xi’an 710064, China; 4 Postdoctoral Workstation, Tibet Tianlu Co., Ltd., Lhasa 850000, China

**Keywords:** road engineering, glass fiber reinforced polymer, asphalt mastic, recycle, wind turbine blade waste

## Abstract

Glass fiber reinforced polymer (GFRP) is the main composite material used in wind turbine blades. In recent years, zero-carbon energy sources such as wind power have been widely used to reduce carbon emissions, resulting in a large amount of waste GFRP, and causing serious environmental problems. To explore efficient ways to recycle waste GFRP, this study explores the impact of adding GFRP powder (nominal maximum particle size ≤ 0.075 mm) on the high and low temperature properties of asphalt mastic. Samples of GFRP asphalt mastics were prepared with filler-asphalt mass ratios of 0.01:1, 0.1:1, 0.8:1, and 1:1, as well as two control samples of limestone filler asphalt mastics with filler-asphalt mass ratios of 0.8:1 and 1:1. The study analyzed the effect of GFRP on the asphalt mastic’s performance using temperature sweep, MSCR, and BBR tests. Results showed that the presence of GFRP improved the high-temperature resistance and recovery of asphalt mastic but led to decreased low-temperature crack resistance. The results suggest that GFRP has the potential to be used as a filler in asphalt mastic, with a recommended filler-asphalt mass ratio range of less than 0.8:1 for optimal low-temperature performance. However, further research is necessary to determine the optimal content of GFRP in asphalt mastic and to study its impact on other road performance metrics.

## 1. Introduction

Greenhouse gas emissions from thermal power generation are a major concern. In 2015, the power sector was responsible for producing approximately 355 million tons of carbon dioxide, accounting for 38% of China’s total carbon emissions from energy consumption [1]. To meet the goal of limiting global climate rise to 1.5 °C and achieving carbon neutrality, the power system is transitioning to zero-carbon sources of energy. As a result, the utilization of zero-carbon sources such as solar and wind energy is increasing; for instance, wind power generation has risen by 31% from 2019 to 2021 [2,3]. However, the widespread use of wind power has also created environmental issues. Wind turbine blades are largely composed of glass fiber-reinforced polymer (GFRP), which is difficult to decompose naturally due to its good mechanical and stable chemical properties [4]. The global output of waste GFRP is estimated to reach 225,000 tons per year by 2034, and the total amount of waste wind power blades will surpass 2 million tons by 2050 [5,6]. As a result, recycling GFRP waste has become a significant and pressing issue. Currently, waste GFRP can be recycled using biological, chemical, or physical methods [7,8].

The methods for recycling Glass Fiber Reinforced Plastic (GFRP) waste are still in the exploratory phase. The biological method is difficult to implement due to a large amount of waste. In contrast, chemical methods, such as pyrolysis and hydrolysis, are costly due to the cross-linked nature of the resin matrix [9,10]. The physical recycling method, which involves breaking down the waste GFRP into powder (nominal maximum particle size ≤ 0.075 mm) or fibers by crushing, grinding, or milling, is the most widely used and has the lowest cost. This method can be used as filler or reinforcement in building materials and has a little environmental impact [11,12].

In the construction industry, asphalt pavement is a common form of pavement used in road projects [13,14,15,16]. The performance of the asphalt mixture depends on the gradation of aggregate and the properties of the asphalt mastic, which is made of asphalt and mineral powder and functions as a bonding agent. The ratio of the filler to the asphalt (in mass) and the type of filler used have been shown to significantly impact the properties of the asphalt mastic. Over-exploitation of natural minerals has led to a shortage of resources and environmental damage, so finding alternative, sustainable materials is important [17,18].

The conclusion of the Strategic Highway Research Program (SHRP) research [19] showed that the impact of asphalt mastic on the high-temperature performance of asphalt mixture is 29%, and on low-temperature performance is 87%. On fatigue, performance is 52% [20]. The study found that the type of filler and the filler-to-asphalt mass ratio significantly affect the properties of asphalt mastic and, therefore, the viscoelasticity and rheology of asphalt mixtures [21,22,23]. One study by Lagos-Varas showed that adding filler increases the stiffness of asphalt and reduces permanent deformation at high temperatures [24]. Another study by Zheng suggested that adding filler increases the low-temperature bond strength of the asphalt binder. However, excessive use of mineral powder can cause agglomeration within the asphalt mastic, which reduces the low-temperature bond strength [25]. Liu’s research found that the asphalt-to-filler mass ratio significantly affects the stiffness of asphalt mastic at different temperatures but has little effect on the low-temperature creep rate [26].

The excessive utilization of natural stones in the construction sector has led to a shortage of resources and environmental degradation, making it imperative to find economical and sustainable substitutes for conventional materials [27,28]. One area for substitution is the use of mineral powders in asphalt mixes, which can be completely or partially replaced by using waste materials such as waste tire rubber powder, slag, ceramic powder, calcareous sand, and volcanic ash [29,30]. Rochlani’s research showed that the porous structure and large specific surface area of ceramic powder are major factors that contribute to the higher stiffness, improved aging characteristics, stronger interaction between the asphalt and filler, and greater resistance to rutting in ceramic powder asphalt mastic [31]. Zhao’s study found that when used in preparation of asphalt mastic, coral reef geotechnical materials provide comparable low-temperature performance but slightly poorer high-temperature performance compared to conventional materials, thereby offering a new possibility for island asphalt pavement construction [32]. Wang’s comparison of high-temperature properties of asphalt mastic with four different fillers revealed that the type of filler has a substantial impact on the optimal range of filler admixture [33]. Despite the environmental and sustainability benefits of using waste as filler in asphalt mixtures, specific parameters must be set for the waste to guarantee minimum road performance.

The feasibility of using crushed waste Glass Fiber Reinforced Polymer (GFRP) as a filler in asphalt mastic was explored in this study. The high and low temperature rheological properties of asphalt mastic containing waste GFRP were compared to those of limestone asphalt mastic, using temperature sweep, multiple stress creep recovery (MSCR), and bending beam rheometer (BBR) tests. The tests were conducted at varying levels of GFRP content, and the results were used to evaluate and propose corresponding high-temperature and low-temperature evaluation indexes. GFRP, composed of epoxy resin and glass fiber, has good mechanical strength and resistance to corrosion, making it a suitable option for use in asphalt mixtures. However, despite its potential as a high-quality fiber modifier, a large amount of waste GFRP produced annually makes this a small solution to a big problem.

## 2. Materials and Methods

### 2.1. Materials

The materials utilized in this study consisted of Penetration Grade 70/100 asphalt, crushed waste GFRP powder, and limestone powder. Both waste GFRP powder and limestone powder were sifted through a 200-mesh sieve. The fundamental attributes of the asphalt are displayed in Table 1, while the key characteristics of the waste GFRP powder and limestone powder are displayed in Table 2.

**Figure 1:**
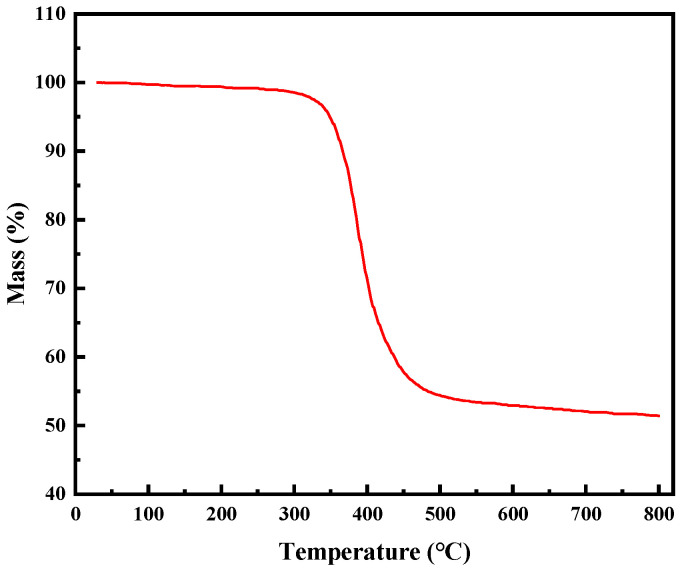
TGA result of the GFRP powder.

GFRP is mainly composed of epoxy resin and glass fibers. Glass fibers have good high-temperature resistance, while the high-temperature resistance of epoxy resin varies depending on the composition of the material. Due to the uncertainty of the composition of epoxy resin in the waste GFRP powder, and the unclear effects of environmental factors on the thermal stability of GFRP during the use of wind turbine blades, TGA analysis was performed on the GFRP to prevent the melting or combustion of the waste GFRP powder during the experimental process and ensure the thermal stability of GFRP material in the HMA mixing process. Figure 1 shows the results of the thermogravimetric analysis conducted on the GFRP powder. It was observed that the mass loss of the GFRP powder occurs primarily between 300 °C and 450 °C, indicating that the decomposition temperature of the epoxy resin is above 300 °C. At 450 °C, the resin has almost completely decomposed, accounting for about 55% of the total mass. The remaining 45% consists of high-temperature-resistant glass fiber and pyrolytic carbon. These results demonstrate that the GFRP powder has excellent thermal stability, making it suitable for asphalt pavement construction.

### 2.2. Sample Preparation Process

The asphalt was heated until it reached a fluid state at 160 °C, while the filler was dried until it reached a constant mass at the same temperature. The GFRP powder and limestone powder were mixed with the asphalt and stirred for 30 min at 160 °C and a speed of 1000 rpm.

Table 3 contains the composition and corresponding labels of all the samples used in this study. The high specific surface area of the filler often results in an uneven dispersion when added to the asphalt mixture. Therefore, to ensure consistent distribution of the filler in the asphalt mastic, fillers with a filler-asphalt mass ratio of 0.8:1 and 1:1 were added in three separate increments.

### 2.3. Methods

#### 2.3.1. Temperature Sweep Test

According to the Strategic Highway Research Program (SHRP) requirements, the temperature sweep test was carried out using a dynamic shear rheometer (DSR) to assess the high-temperature rheological properties of the GFRP asphalt mastic. The test involves simulating the load of a vehicle on the asphalt pavement. The test results produce two important parameters, the complex modulus G* and the phase angle δ, which describe the rheological properties of the asphalt mastic. The complex modulus G* represents the resistance of the asphalt material when subjected to shear. The greater the G*, the better the high-temperature performance of the asphalt mastic at the same temperature. On the other hand, the phase angle δ reflects the viscoelastic properties of the asphalt. A low value of δ indicates that the asphalt material has a higher ability to recover a large portion of its elastic deformation, which makes it better at resisting high-temperature rutting. The index G*/sin δ, put forward by SHRP, is used to evaluate and control the resistance of asphalt mastic to rutting at high temperatures. The index can also calculate the continuous grading for high temperatures. Finally, the complex viscosity η* reflects the fluidity of the asphalt mastic at different temperatures, which can be seen as an indicator of the workability of the asphalt mixture during construction. The test was performed using a 25 mm diameter parallel plate mold with a 1 mm plate spacing and a fixed frequency of 10 ± 0.1 rad/s. During the trial, measurements of the phase angle (δ), complex shear modulus (G*), and complex viscosity (η*) of each sample were taken at temperatures of 46 °C, 52 °C, 58 °C, 64 °C, 70 °C, 76 °C, and 82 °C. Each sample was tested three times.

#### 2.3.2. MSCR Test

The MSCR tests in this study were carried out per the ASTM D7405-15 standard [35]. A 25 mm diameter test plate mold with a parallel plate spacing of 1 mm was used. The MSCR tests were performed at 60 °C with two stress levels, 0.1 kPa and 3.2 kPa. Each sample was tested three times. The samples underwent 20 cycles of loading and recovery at a stress level of 0.1 kPa, followed immediately by 10 cycles of loading and recovery at a stress level of 3.2 kPa, with no interval between the two phases. During the loading cycle, the sample was loaded at a constant stress for 1 s. Then the load was removed for 9 s to allow the sample to recover. The irreversible creep compliance *J_nr_* (Equation (1)) and recovery rate R (Equation (2)) reflecting the rutting resistance potential of asphalt can be obtained through the MSCR test. The average values of *J_nr_* and *R* for asphalt samples under 10 stress cycles of 0.1 kPa (*J_nr_*_0.1_ and *R*_0.1_) and the average values of *J_nr_* and *R* for asphalt samples under 10 stress cycles of 3.2 kPa, (*J_nr_*_3.2_ and *R*_3.2_) were calculated respectively according to Equations (3)–(6).

(1) $$R=\frac{\varepsilon_{p}-\varepsilon_{u}}{\varepsilon_{p}}\times 100\%$$

(2) $$J_{nr}=\frac{\varepsilon_{u}}{\sigma }$$

where $\varepsilon_{p}$ is the peak strain, $\varepsilon_{u}$ is the residual strain that cannot be recovered at the end of the recovery stage, and σ is the corresponding stress level applied by the two stages.

(3) $$J_{nr0.1}=\frac{SUM\left[J_{nr}\left(0.1,N\right)\right]}{10}\;\left(N=11\sim 20\right)$$

(4) $$J_{nr3.2}=\frac{SUM\left[J_{nr}\left(3.2,N\right)\right]}{10}\;\left(N=1\sim 10\right)$$

(5) $$R_{0.1}=\frac{SUM\left[\varepsilon_{r}\left(0.1,N\right)\right]}{10}\;\left(N=11\sim 20\right)$$

(6) $$R_{3.2}=\frac{SUM\left[\varepsilon_{r}\left(0.1,N\right)\right]}{10}\;\left(N=1\sim 10\right)$$

#### 2.3.3. BBR Test

The results of the BBR test are important in evaluating the low-temperature performance of modified GFRP asphalt mastic, as they provide information on the material’s stiffness modulus (S) and creep rate (m). These parameters indicate the material’s ability to resist deformation under low temperature bending loads, which is essential for ensuring the durability and stability of the asphalt pavement. The BBR test was performed by applying a constant load at the mid-span position of the beams and measuring the deformation data with displacement sensors. The test results were obtained at 60 s and temperatures of −12 °C and −18 °C, providing valuable insights into the low-temperature behavior of modified GFRP asphalt mastic. Each sample was tested three times.

## 3. Results

### 3.1. High-Temperature Rheological Performance

**Figure 2:**
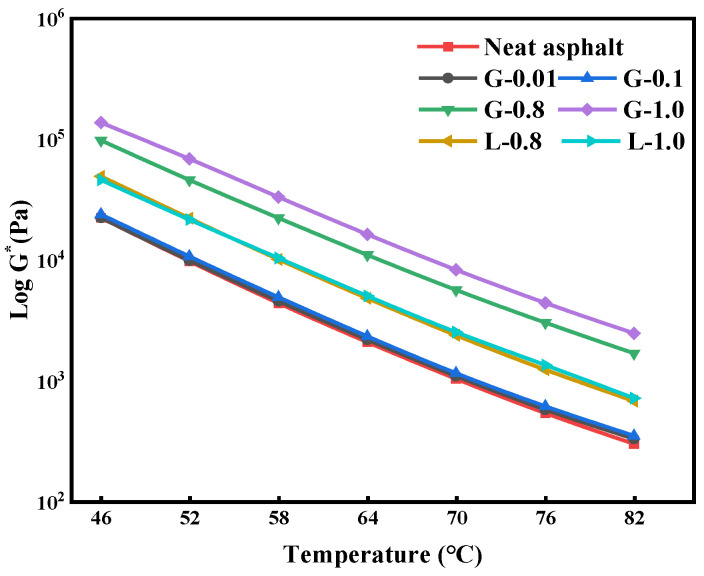
Dynamic shear moduli of asphalts and mastics.

Figure 2 depicts the temperature-log G* graphs for various asphalt mastics. As per the figure, a linear relationship exists between temperature and logarithm of G*. The high temperature performance of asphalt mastic is significantly impacted by the type and quantity of filler. The higher the filler content, the more significant the improvement in the resistance to high-temperature rutting of the asphalt mastic [36,37]. The high-temperature performance of G-1.0 and G-0.8 has been significantly improved, followed by L-1.0 and L-0.8. Conversely, there is no significant change in the high-temperature performance of G-0.01 and G-0.1. Based on the material properties, GFRP has a larger surface area and can absorb more free asphalt compared to limestone powder, making it more effective in enhancing the high-temperature performance of the asphalt mastic compared to limestone powder of the same content.

**Figure 3:**
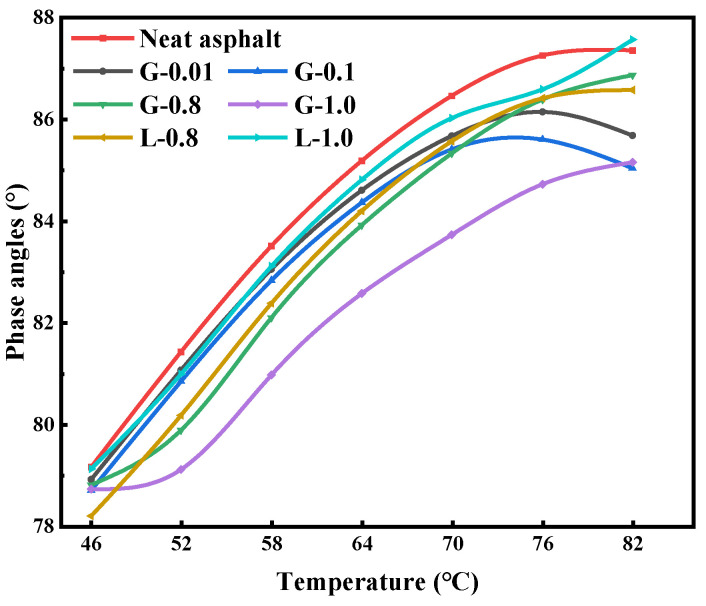
The phase angles of asphalts and mastics.

As shown in Figure 3, the phase angle δ displays an increasing and decreasing pattern as the temperature increases. The addition of a high amount of GFRP powder (filler-asphalt mass ratio ≥ 0.8:1) significantly reduces the δ of the asphalt mastic, demonstrating that GFRP powder gives the asphalt mastic more elasticity, thereby increasing its proportion of elastic deformation during deformation, which results in a reduction of the permanent deformation of the asphalt mastic at high temperatures. Limestone powder can also provide some elasticity to the asphalt mastic, and its effect is comparable to G-0.01 and G-0.1.

As demonstrated in Table 4, the results of the continuous grading temperature calculations show that compared to neat asphalt. The asphalt mastic has improved rutting resistance. The addition of small amounts of GFRP powder (filler-asphalt mass ratio ≤ 0.1:1) does not have a significant effect on the continuous grading high temperature (0.7 °C~2.3 °C). As the content of GFRP powder increases, the continuous grading high temperature also increases gradually. The continuous grading temperature of G-0.8 has seen a nearly 20% improvement. While large amounts (filler-asphalt mass ratio ≥ 0.8:1) of limestone powder can also improve the high-temperature performance of neat asphalt, its effect is not as strong as that of GFRP in asphalt mastic.

**Figure 4:**
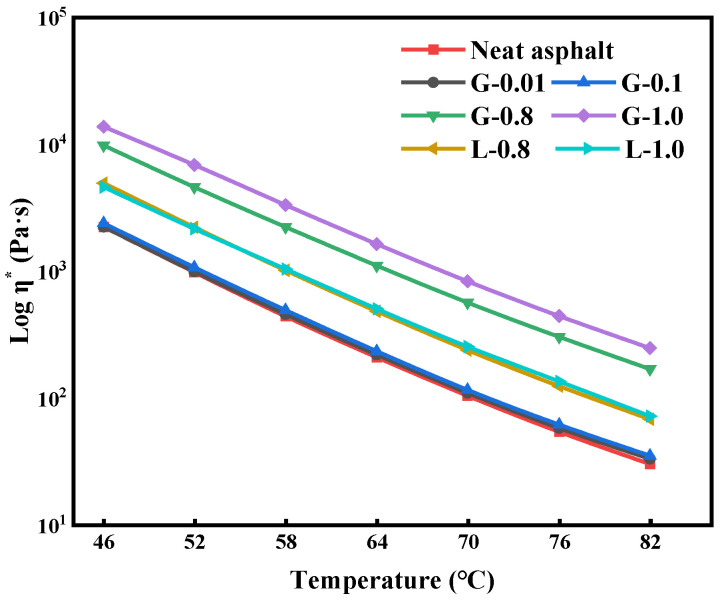
Complex viscosity of asphalts and mastics.

As seen in Figure 4, there is a clear linear relationship between log η* and temperature. The value of η* decreases gradually with the increase in temperature due to the enhanced movement between asphalt molecules, which makes it easier for the asphalt to be displaced under external forces. During the temperature range of 46 °C to 82 °C, the complex viscosity of GFRP asphalt mastic is higher than that of limestone asphalt mastic. This is due to the stronger interaction between GFRP and asphalt, which improves the complex viscosity of the asphalt mastic and enhances its resistance to rutting at high temperatures. The η* value increases with the increase of GFRP content because the larger surface area of GFRP provides more contact with the asphalt binder, leading to a stronger interaction between the two materials and an increase in the structural asphalt content in the GFRP asphalt mastic. However, it may be necessary to choose a higher construction temperature to ensure the fluidity of the asphalt mixture, as GFRP asphalt mastic requires a temperature increase of 6 °C to 12 °C to achieve the same η* as conventional limestone asphalt mastic.

### 3.2. High-Temperature Creep and Recovery Behavior

**Figure 5:**
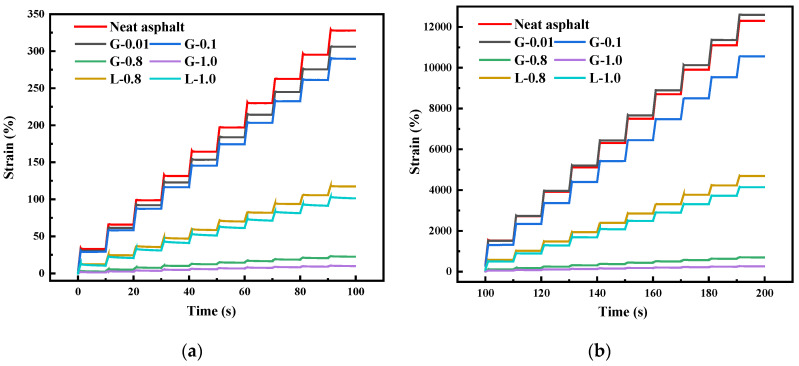
Strain-time diagram of asphalts and mastics: (**a**) 0.1 kPa; (**b**) 3.2 kPa.

Figure 5 illustrates the MSCR time-strain curves of different asphalt mastics. It can be observed that the inclusion of filler reduces the shear strain of the asphalt mastic and enhances its resistance to deformation. The higher the content of the filler, the better the rutting resistance of the asphalt mastic. This is likely because, with an increase in the filler content, the particle-particle interaction and filler-asphalt interaction start to dictate the rheological behavior of the asphalt mastic, thereby reducing the free asphalt in the mastic. Additionally, it can be seen that the change in shear strain of the asphalt mastic containing GFRP powder is smaller compared to that of limestone powder, indicating that its stress sensitivity is lower and that GFRP powder can better enhance the permanent deformation resistance of the asphalt mastic. However, it should be noted that not all contents of GFRP powder can improve the high-temperature performance of asphalt mastic. For example, g-0.01 undergoes a greater shear strain under high stress.

**Figure 6:**
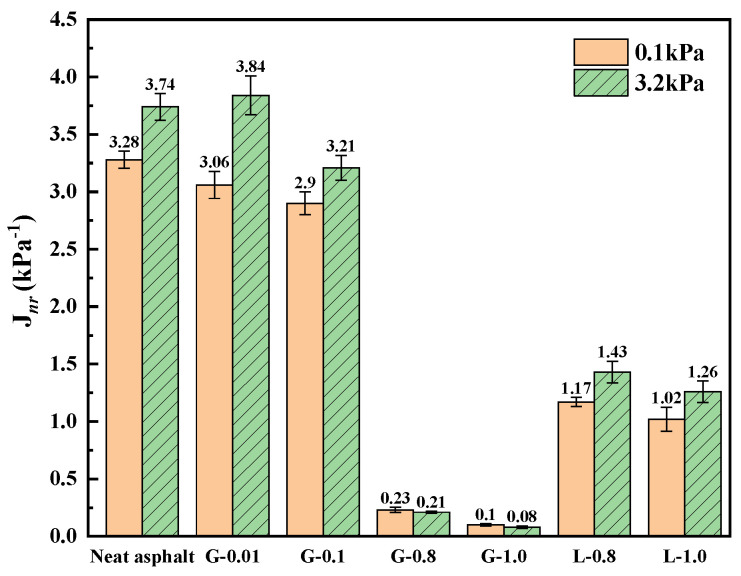
*J_nr_* of asphalts and mastics at 60 °C.

The non-recoverable creep compliance (*J_nr_*), which represents the rutting sensitivity, and the recovery rate (*R*), which represents the elastic response, of asphalt mastic were obtained through the MSCR method [35]. As shown in Figure 6, the *J_nr_* of the asphalt mastic decreases continuously with the increasing content of GFRP powder. At a content of 0.8, there is a sharp decrease in *J_nr_*. Compared to limestone filler, GFRP powder has a better ability to inhibit the deformation of the asphalt mastic, resulting in a permanent deformation that is 80.3% to 93.6% lower than that of limestone asphalt mastic. This is likely due to the strong mechanical properties of GFRP powder and its effective combination with asphalt through physical and chemical interactions. The *J_nr_* at 3.2 kPa shows a similar trend to that at 0.1 kPa, with a rapid decrease when the filler-asphalt mass ratio t reaches 0.8:1. The difference between *J_nr_*_0.1_ and *J_nr_*_3.2_ also narrows. However, there is a slight deterioration of *J_nr_* when the filler-asphalt mass ratio is 0.01:1.

**Figure 7:**
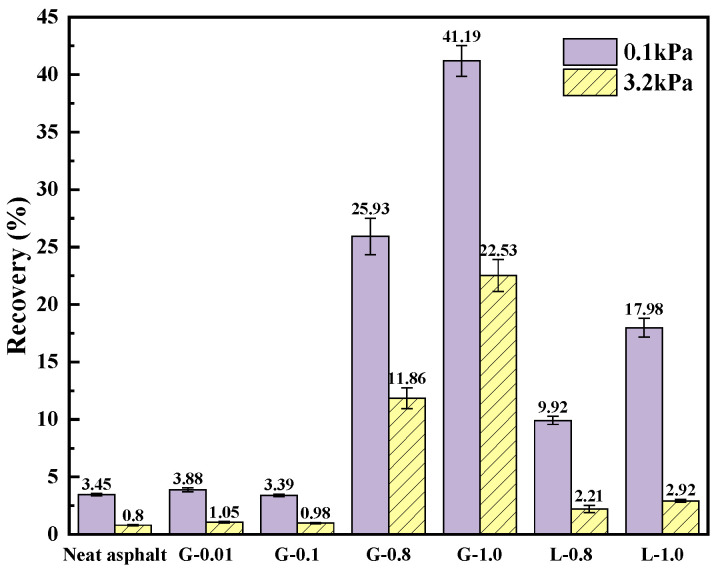
R% of asphalts and mastics at 60 °C.

It is evident from Figure 7 that for a filler-asphalt mass ratio less than 0.1:1, the addition of GFRP powder has no substantial impact on the recovery rate of asphalt mastic deformation. However, when the filler-asphalt mass ratio reaches 0.8:1, the R-value of the asphalt mastic increases with the increase of the filler content. This trend can also be observed in the case of the lime powder filler. This suggests that with an increase in the filler, the viscosity of asphalt mastic decreases, and its adhesive becomes more elastic. It is also observed that the recovery rate at 0.1 kPa is significantly higher than that at 3.2 kPa, which is particularly noticeable in the case of limestone asphalt mastic, indicating its sensitivity to stress. Under both 0.1 kPa and 3.2 kPa load levels, the recovery rate of the GFRP powder asphalt mastic is higher compared to that of the limestone asphalt mastic, reaching as high as 7.7 times at 3.2 kPa. This highlights that GFRP powder can significantly improve the high-temperature deformation recovery ability of asphalt mastic under high stress.

From the change of *J_nr_* and *R*, it can be seen that the content and type of filler greatly impact the stress sensitivity of asphalt mastic. Therefore, *J_nrdiff_* (Equation (7)) and *J_nrslope_* (Equation (8)) is used to study the sensitivity of asphalt mastic.

(7) $$J_{nrdiff}=\frac{J_{nr3.2}-J_{nr0.1}}{J_{nr0.1}}\times 100$$

(8) $$J_{nrslope}=\frac{J_{nr3.2}-J_{nr0.1}}{3.2-0.1}\times 100$$

To quantify the hardening effect of fillers on asphalt mastic, the High-temperature Stiffening Index (HIS)(Equation (9)) is proposed. The smaller *J_nr_* and *J_nrslope_* mean smaller permanent deformation and lower stress sensitivity of asphalt mastic. Therefore, the ratio of *J_nr_* and *J_nrslope_* can more comprehensively evaluate the rutting resistance of asphalt mastic [38].

(9) $$HSI=\log\left|\frac{J_{nr3.2-virgin}}{J_{nr}{}_{3.2-stiffened}}\times \frac{J_{nrslope-virgin}}{J_{nrslope-stiffened}}\right|$$

Table 5 shows that the difference between *J_nrdiff_* and *J_nrslope_* is negligible for asphalt mastic with a filler-asphalt mass ratio less than 0.1:1. However, as the filler content increases, *J_nr_*_0.1_ decreases and a noticeable difference between *J_nrdiff_* and *J_nrslope_* can be observed. Sorting the stress sensitivity of asphalt mastic by *J_nrdiff_* leads to results that are not in line with the actual performance, particularly for G-0.8 and G-1.0 asphalt mastics with the lowest *J_nr_*_0.1._ In these cases, *J_nrdiff_* values are exaggerated and don’t accurately reflect the performance of the asphalt mastic.

On the other hand, the behavior of *J_nrslope_* aligns with the actual hardening condition of the asphalt mastic. Therefore, it is more suitable for evaluating the stress sensitivity of asphalt mastic with low *J_nr_* values and high stiffness. The results indicate that GFRP filler reduces the stress sensitivity of the asphalt mastic better than limestone filler. Furthermore, when the filler-asphalt mass ratio exceeds 0.8:1, the permanent deformation of the asphalt mastic is similar under 0.1 kPa and 3.2 kPa load levels of GFRP asphalt mastic, indicating that the stress level has no significant impact on the permanent deformation. As a result, the calculated *J_nrslope_* is close to zero.

It is evident from the results of the stress sensitivity evaluation that *HSI* reflects the same trend as the stress sensitivity analysis and *J_nr_* results, effectively capturing the hardening behavior of asphalt mastic. A small amount of GFRP powder (filler-asphalt mass ratio ≤ 0.01:1) has a negative impact on the permanent deformation performance of asphalt mastic, as indicated by a negative *HSI* value. On the other hand, the highest and second highest *HSI* values were observed in G-1.0 and G-0.8, respectively. This implies that the higher the *HSI* value, the better the high-temperature rutting resistance of the asphalt mastic. *HSI* combines *J_nr_* and *J_nrslope_* to provide a comprehensive characterization of the hardening degree and stress sensitivity of asphalt mastic.

### 3.3. Low-Temperature Rheological Performance

**Figure 8:**
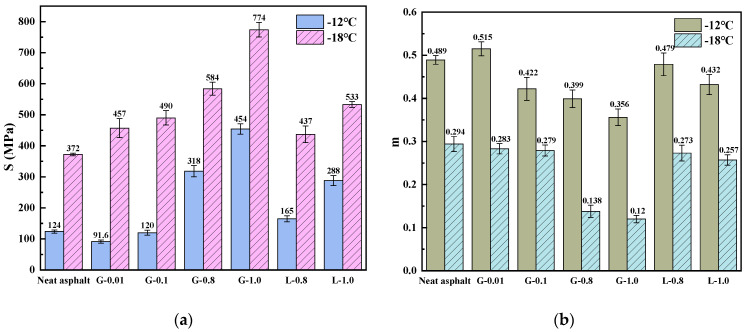
BBR test results in terms of S and m of asphalts and mastics: (**a**) Stiffness (S); (**b**) Creep rate (m).

The results of the low-temperature performance evaluation indicate that the addition of GFRP powder has a negative impact on the low-temperature performance of asphalt mastic. As shown in Figure 8, with increasing GFRP powder content, the stiffness modulus (S) of the asphalt mastic increases while the creep rate (m) decreases. This results in a decrease in the low-temperature grade of the GFRP asphalt mastic, from −22 °C to −16 °C as the filler-asphalt mass ratio increases from 0.1:1 to 1:1. On the other hand, limestone filler with the same content retains the low-temperature grade of the neat asphalt. However, it should be noted that the low-temperature performance of G-0.01 is slightly improved, which could be attributed to the dispersion form of the GFRP powder in the asphalt and physical and chemical reactions.

The larger the difference between *T*_*C*,*S*_ and *T*_*C*,*m*_, the more imbalanced the low-temperature performance of the binder is, and the more likely it is to crack. As shown in Figure 8, the difference between *T*_*C*,*S*_ and *T*_*C*,*m*_ of the asphalt mastic with GFRP powder increases with the increase of GFRP content, indicating the low-temperature performance of the asphalt mastic becomes more and more imbalanced, and the risk of cracking increases accordingly. However, asphalt mastic with limestone filler has a relatively stable and balanced low-temperature performance. Adding GFRP powder improves the low-temperature performance of the G-0.01, but the improvement effect is insignificant. The continuous low-temperature classification results show that the *T*_*C*,*S*_, *T*_*C*,*m*_ and Δ*T_C_* values calculated by Equations (10) and (11) can accurately reflect the low-temperature performance of the asphalt mastic, providing a more comprehensive evaluation method for low-temperature cracking resistance of the asphalt binder.

(10) $$T_{C,S}=T_{1}+\frac{\left(T_{1}-T_{2})(\log300-\log S_{1}\right)}{\log S_{1}-\log S_{2}}-10$$

(11) $$T_{C,m}=T_{1}+\frac{\left(T_{1}-T_{2})(0.3-m_{1}\right)}{m_{1}-m_{2}}-10$$

where *T*_*C*,*S*_ and *T*_*C*,*m*_ are the critical temperatures controlled by *S* and *m* values; *S*_1_ and *S*_2_ are the creep stiffness at temperatures *T*_1_ and *T*_2_; *m*_1_ and *m*_2_ are the creep rates at temperatures *T*_1_ and *T*_2_; *T*_1_ and *T*_2_ are the temperatures at which *S* or *m* values pass or fail the standard.

It can be observed from Table 6 that the addition of fillers increases the critical temperature of the asphalt mastic, indicating a negative impact on its low-temperature performance. The increase in the critical temperature (*T*_*C*,*S*_) is more pronounced with the increase in filler content. All samples show a positive Δ*T_C_* value, indicating that the low-temperature hardening causes a loss of elasticity and is the primary cause of the low-temperature cracking of the binder. The decrease in critical temperature of GFRP asphalt mastic is much more significant compared to that of limestone asphalt mastic. The low-temperature performance of L-0.8 is comparable to G-0.1. The Δ*T_C_* of G-1.0 even reaches 6.08, indicating a higher stiffness at low temperatures and a longer stress relaxation time, making it more susceptible to brittle cracking. This may be due to GFRP powder absorbing excessive amounts of free asphalt, disrupting the balance of the asphalt mastic system, and leading to a reduction in the asphalt phase in the mastic, which ultimately increases the low-temperature stiffness of the asphalt mastic.

It can be found that *T*_*C*,*S*_ are more sensitive to the filler incorporation by comparing different asphalt mastics, so *S* determines the critical temperature of asphalt mastic. To quantify the effect of filler on low-temperature performance, the low-temperature hardening index *LSI_S_* (Equation (12)) based on *T*_*C*,*S*_ is proposed, and *LSI_m_* (Equation (13)) based on *T*_*C*,*m*_ is calculated as the control [39].

(12) $$LSI_{S}=\frac{T_{c,S-virgin}-T_{c,S-stiffened}}{T_{c,S-virgin}}\times 100\%$$

(13) $$LSI_{m}=\frac{T_{c,m-virgin}-T_{c,m-stiffened}}{T_{c,m-virgin}}\times 100\%$$

**Figure 9:**
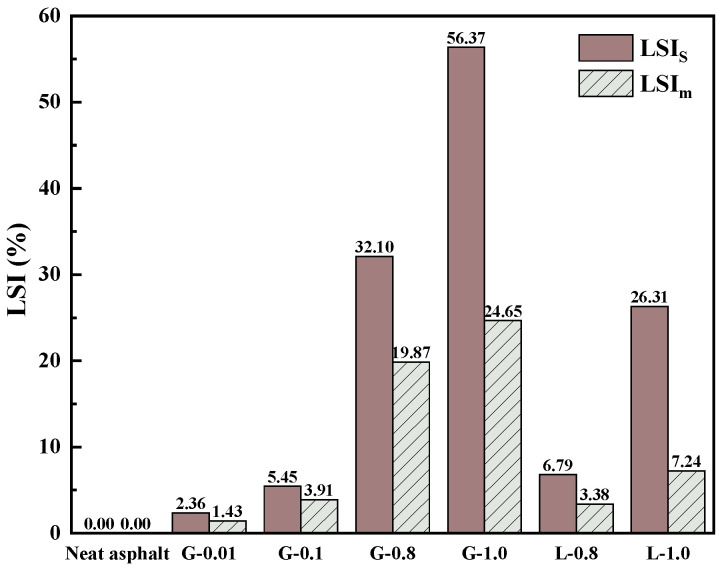
*LSI_S_* and *LSI_m_* of different samples.

**Figure 10:**
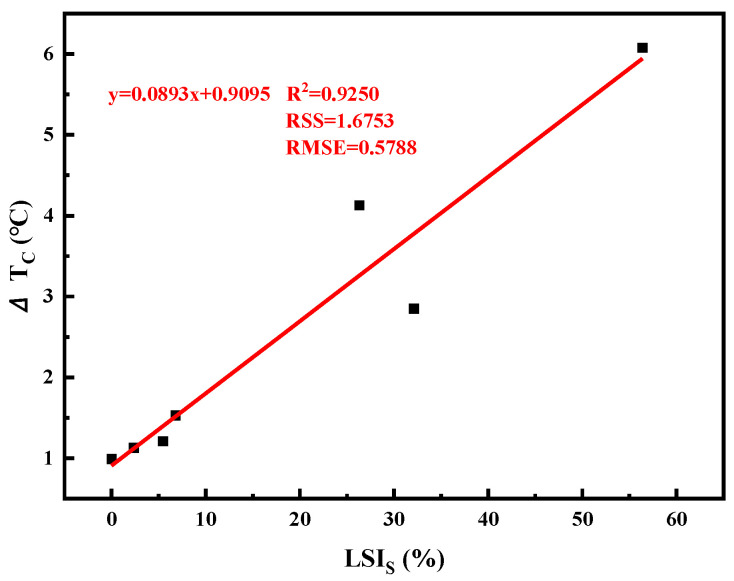
Correlation between *LSI_S_* and Δ*T_C_*.

It can be seen from Figure 9 that *LSI_S_* and *LSI_m_* show a similar trend, both of which can evaluate the low-temperature performance of asphalt mastic. *LSI_S_* is more suitable for asphalt mastic with stiffness failure as the main evaluation index. Figure 10 shows that *LSI_S_* has a high correlation with Δ*T_C_*, which confirms that *LSI_S_* can reflect the hardening degree of asphalt and asphalt mastic at low temperatures. *LSI_S_* results also showed that GFRP powder played an adverse role in the low temperature hardening process of the mastic.

## 4. Conclusions

This study aimed to investigate the impact of GFRP powder on the high and low-temperature rheological properties of asphalt mastic. Samples of GFRP asphalt mastic with filler-asphalt mass ratios of 0.01:1, 0.1:1, 0.8:1 and 1:1, and limestone asphalt mastic with filler-asphalt mass ratios of 0.8:1 and 1:1 were prepared. The results of temperature sweep tests, MSCR tests, and BBR tests were analyzed to determine the effect of GFRP powder on the high and low temperature performance of the asphalt mastic. The following conclusions and recommendations can be drawn from this study:

(1) The composition and physical characteristics of GFRP lead to improved high-temperature deformation resistance and recovery but also result in reduced low-temperature crack resistance.
(2) *J_nrslope_*, in addition to *J_nrdiff_*, provides a more accurate evaluation of the stress sensitivity of the asphalt mastic at high temperatures. Therefore, two indices were proposed to evaluate the hardening degree of the high and low temperature asphalt mastic, considering the influence of fillers on its properties.
(3) The results of this study indicate the potential of GFRP powder as a pavement filler, with improved high-temperature performance compared to limestone filler. However, it is recommended to limit the filler-asphalt ratio of GFRP asphalt mastic to below 0.8:1 to ensure optimal low-temperature performance.
(4) While this study provides valuable insights into using GFRP powder as a pavement filler, further research is needed to determine the optimal content of GFRP filler and better understand the interaction mechanism between GFRP powder and asphalt.

## Figures and Tables

**Table 1:** Basic Properties of Penetration Grade 70/100 Asphalt.

Item	Method [34]	Test Value
Penetration (25 °C, 0.1 mm)	T 0604	78
Softening point (°C)	T 0606	47
Ductility (5 °C, cm)	T 0605	62.5
Viscosity(60 °C)	T 0620	235

**Table 2:** Basic Properties of Waste GFRP Powder and Limestone Powder.

Item		GFRP	Limestone
Density (g/cm^3^)		2.37	2.65
Specific surface area (SSA) (m^2^/g)		2.15	1.95
Particle size range (%)	≤0.6 mm	100	100
	≤0.3 mm	96.5	100
	≤0.075 mm	92.3	98.6
Moisture content (%)		0.55	0.32

**Table 3:** Codes of asphalt mastics.

Asphalt Mastic Code	Filler-Asphalt Mass Ratio	Filler
G-0.01	0.01:1	GFRP
G-0.1	0.1:1	GFRP
G-0.8	0.8:1	GFRP
G-1.0	1.0:1	GFRP
L-0.8	0.8:1	Limestone
L-1.0	1.0:1	Limestone

**Table 4:** Continuous grading high temperature.

	Neat Asphalt	G-0.01	G-0.1	G-0.8	G-1.0	L-0.8	L-1.0
Continuous grading temperatures/°C	65.5	66.2	67.8	78.1	82.5	73.2	76.7

**Table 5:** *J_nrdiff_*, *J_nrslope_* and high temperature stiffening index of different samples.

	Neat Asphalt	G-0.01	G-0.1	G-0.8	G-1.0	L-0.8	L-1.0
*J_nrdiff_*	14.15	25.51	10.78	−6.38	−20.10	21.62	24.19
*J_nrslope_*	14.96	25.18	10.07	−0.47	−0.64	8.19	7.93
*HSI*	0	−0.24	0.24	2.75	3.04	0.68	0.75

**Table 6:** Critical temperatures of different samples.

	*T_C,S_* (°C)	*T_C,m_* (°C)	Δ*T_C_* (°C)
Neat asphalt	−26.83	−27.82	0.99
G-0.01	−26.43	−27.56	1.13
G-0.1	−25.91	−27.12	1.21
G-0.8	−21.42	−24.28	2.85
G-1.0	−17.34	−23.42	6.08
L-0.8	−25.68	−27.21	1.53
L-1.0	−22.40	−26.53	4.13

## Data Availability

The data presented in this study are available on request from the corresponding author.
